# Development of a strategic plan for food security and safety in the Inuvialuit Settlement Region, Canada

**DOI:** 10.3402/ijch.v73.25091

**Published:** 2014-08-08

**Authors:** Myriam Fillion, Brian Laird, Vasiliki Douglas, Linda Van Pelt, Diane Archie, Hing Man Chan

**Affiliations:** 1 Centre for Advanced Research in Environmental Genomics, University of Ottawa, Ottawa, ON, Canada; 2 School of Public Health and Health Systems, University of Waterloo, Waterloo, ON, Canada; 3 School of Health Sciences, University of Northern British Columbia, Prince George, BC, Canada; 4 Inuvialuit Regional Corporation, Inuvik, NT, Canada

**Keywords:** food security, health, Inuit, Inuvialuit Settlement Region, workshop, planning, participatory approach

## Abstract

**Background:**

Current social and environmental changes in the Arctic challenge the health and well-being of its residents. Developing evidence-informed adaptive measures in response to these changes is a priority for communities, governments and researchers.

**Objectives:**

To develop strategic planning to promote food security and food safety in the Inuvialuit Settlement Region (ISR), Northwest Territories (NWT), Canada.

**Design:**

A qualitative study using group discussions during a workshop.

**Methods:**

A regional workshop gathered Inuit organizations and community representatives, university-based researchers from the Inuit Health Survey (IHS) and NWT governmental organizations. Discussions were structured around the findings from the IHS. For each key area, programs and activities were identified and prioritized by group discussion and voting.

**Results:**

The working group developed a vision for future research and intervention, which is to empower communities to promote health, well-being and environmental sustainability in the ISR. The group elaborated missions for the region that address the following issues: (a) capacity building within communities; (b) promotion of the use of traditional foods to address food security; (c) research to better understand the linkages between diseases and contaminants in traditional foods, market foods and lifestyle choices; (d) and promotion of affordable housing. Five programs to address each key area were developed as follows: harvest support and traditional food sharing; education and promotion; governance and policy; research; and housing. Concrete activities were identified to guide future research and intervention projects.

**Conclusions:**

The results of the planning workshop provide a blueprint for future research and intervention projects.

Over the past decades, the Arctic has been experiencing a series of changes in its ecological, social, cultural, political and economic systems (1). These changes are affecting all segments of life, health and well-being in the Arctic. In addition, a recent report published by the Council of Canadian Academies (CCA) on the state of knowledge on Aboriginal food security in northern Canada concluded that the current food security crisis has long-term implications for the health and well-being of Inuit communities (2).

Health and well-being in the Canadian Arctic have been extensively investigated during the International Polar Year Inuit Health Survey: Inuit Health in Transition and Resiliency (IHS) (3). The survey was developed with local stakeholders to gain a better understanding of the factors contributing to Inuit health and health transition, in order to find solutions for these Inuit-specific challenges and to inform policy makers (4). The design and methods of the IHS are described elsewhere (4). The IHS was a comprehensive cross-sectional health survey carried on in late summer and fall of 2007 and 2008 in 3 jurisdictions of the Canadian Inuit Nunangat: the Inuvialuit Settlement Region (ISR – Northwest Territories), Nunavut and Nunatsiavut (Labrador).

This paper reports the findings of a workshop in the ISR, located in the western Canadian Arctic, in the northerly portion of the Northwest Territories (NWT) and the North Slope of the Yukon Territory (5). The region comprises 6 communities: Inuvik, Aklavik, Tuktoyaktuk, Sachs Harbour, Ulukhaktok and Paulatuk, and has a population of 5,777 inhabitants, of which half is Inuit (6). In the ISR, a total of 288 households and 362 individuals, aged 18 years and older, participated in the IHS. The final report is available online (http://www.irc.inuvialuit.com/publications/pdf/ihs-report-final.pdf). The main results from the IHS show that key themes were nutrition transition, food security, chronic diseases and contaminants.

Inuit are currently going through a nutritional transition, which is reflected by a decrease in the consumption of traditional food and an increase in the consumption of low nutritional value market food (7). Recently, food insecurity has been documented in the Inuit communities (7, 8). Results from the IHS show that Inuit from the Canadian Arctic disproportionately experience food insecurity compared to the general Canadian population: 62.6% of the Inuit households are food insecure, 27.2% of them being severely food insecure (9), compared to 7.7% of the Canadian households according to the 2007–2008 Canadian Community Health Survey (10). This poses a threat to Inuit health because of the clear relationship between food insecurity and poorer health (8).

In addition, nutrition transition and food insecurity have been associated with nutrient deficiencies and to the emergence of nutrient deficiency-related health issues (7). The link between nutrient deficiency and increased caloric intake has also been documented in the Inuit population. Chronic diseases, especially diabetes and obesity, are emerging in the Inuit population (1, 11).

Since the 1980s, environmental contaminants, such as heavy metals and persistent organic pollutants (POPs), have been identified in all components of the Arctic ecosystem (12). The identification of these contaminants in traditional food has been associated with a reduced confidence in food safety, even if the consumption of traditional foods has proven to provide important health benefits (13).

These issues were discussed during a regional workshop, in July 2012, in Inuvik, NWT, Canada. The objectives of this paper are to describe the process followed during the regional workshop and to provide recommendations regarding future research and intervention to improve health and well-being in the ISR, NWT, Canada.

## Materials and methods

The Inuvialuit Regional Corporation (IRC), in collaboration with the key researchers of the Inuit Health Survey (IHS), organized a regional workshop. The goal of this workshop was to develop a multi-stakeholder participatory approach to discuss food security-related health issues in the ISR. Participants to the workshop were from organizations previously involved in the IHS, either as steering committee members or as advisors to the steering committee, as well as regional and territorial health organizations. A group of researchers, Inuvialuit community representatives, as well as local and territorial government organizations stakeholders gathered to discuss the results of the 2007–2008 IHS regarding food safety and food security issues as well the promotion of Inuit health. Ethics approval was obtained from the Research Ethics Boards of the University of Ottawa and the University of Northern British Columbia.

A 2-day workshop was held in Inuvik, NWT, Canada, in July 2012. On the first morning, researchers from the University of Ottawa, University of Northern British Columbia and University of Toronto presented the main findings from the IHS. Discussions directed at specific issues linked to the presentation of the results followed the presentation.

In the afternoon, the participants were asked to form 3 discussion groups, sorted according to their individual areas of expertise, and answered the following questions:

- Vision: What is our goal, what do we want to achieve?
- Mission: How would we achieve these goals?
- Activities and programs: What are the initiatives, ideas and activities that could be implemented to fulfil our missions?

The groups had a brainstorming session addressing each question and presented their results back to all the participants. After all groups presented their input on questions 1 and 2, the vision and mission were developed following a common agreement on the elements that these should include. After brainstorming on the third question, participants presented their suggestions of programs and used colour-coded stickers to prioritize them:

- Yellow: Practical activities to implement
- Pink: Important activities
- Blue: Favourite activities

At the end of the first day, researchers collected all the working documents and collated the most popular ideas under global themes and key areas of focus that emerged from the afternoon discussions. These themes and key areas of focus were presented to the participants the following day. The activities and programs under each theme that received the majority of votes using the stickers were presented to the group, and the whole group discussed details and clarifications. The group then provided final approval for the priority programs and activities.

## Results

### Workshop participants

A total of 23 people participated in this regional workshop. The participants were from different organizations from the ISR communities, local and territorial government organizations and universities (Table I).

**Table I:** Affiliations of the participants to the regional workshop

Categories of stakeholders	Organizations
ISR community representatives	Hunters and Trappers Committees (HTC) Community Corporations (CC)
Local and territorial governments	Inuvialuit Regional Corporation (IRC) Beaufort Delta Health and Social Services Regional Nutritionists NT Deputy Chief Medical Health Officer
National Inuit organization	Inuit Tapiriit Kanatami (ITK)
Researchers	University of Ottawa University of Toronto University of Northern British Columbia Institute for Circumpolar Health Research

### Vision

Group discussions during the workshop led to the development of the following vision for the promotion of health in the ISR: “To empower communities to promote health, well-being and environmental sustainability in the Inuvialuit Settlement Region.”

### Missions

After the formulation of the common vision for the future of health research and intervention in the ISR, the workshop participants identified the following missions to achieve this vision. Mission statements were developed in 4 areas.

#### Capacity building

Participants mentioned that capacity should be built and strengthened within communities. Capacity building should include the development of programs tailored to each community and hands-on educational programs. New initiatives should expand on existing programs and encourage youth to participate in management boards.

#### Promotion of traditional foods

Participants emphasized the need to address food security and retain the quality of the Inuvialuit diet. To achieve this, they recommended to evaluate the existing wildlife management programs combining traditional and scientific knowledge and to investigate healthy and culturally acceptable substitutions for when traditional food is restricted.

#### Research

Discussions in the workshop recognized the need to better understand the linkages between diseases and contaminants in traditional foods, market foods and lifestyle choices. Participants identified further research should identify gaps in knowledge, and research results should be made accessible through online archive or repository of traditional knowledge.

#### Promotion of affordable housing

There was an agreement among the participants that the high costs of housing were a major obstacle to achieve food security. The participants suggested the development of subsidized housing programs to allow increased access to secure housing, and to households to allocate more income to improve food security.

### Activities and programs

Participants identified the following programs and activities that, if implemented would support the vision and achieve the missions mentioned above. Activities and programs are presented under the following 5 themes.

#### Harvest support and traditional food sharing

Participants agreed that programs to provide support to individuals, families and communities willing to harvest traditional foods and to share them should be implemented. These programs could include the following elements:

- Sharing traditional food via food mail between communities could be organized and managed by the Hunters and Trappers Committees (HTCs) and/or explore the possibility of obtaining reduced freight rate as in-kind contributions from local airlines.
- HTCs and/or Community Corporations (CCs) could coordinate the sharing of traditional food within each community.
- Annual and/or seasonal community hunts could be organized in order to supply traditional food to the community.
- A regional processing plant of muskox and/or reindeer meat could be promoted in Inuvik.
- HTCs and/or CCs could coordinate the rental of hunting supplies and equipment and the donation of a part of the harvest to a community freezer or people with limited access to traditional foods.
- Community gardens could be developed in order to grow fresh vegetables for community members during the summer.
- Manufacturers and suppliers could be encouraged to provide support to the community members willing to engage in traditional food harvesting (e.g. Bombardier, Le Baron, Yamaha, Remington).
- A walk-in community freezer could be constructed and/or maintained in each community in order to provide a collective storage for the traditional food.

#### Education and promotion

Participants recognized that education is a key element to empower communities. Adequate health and wellness promotion programs were also identified as key factors in educating individuals, families and communities. Concretely, education and promotion programs and activities could include the following:

- Programs in which elders teach community members how to identify local vegetation could be developed (e.g. mushrooms, wild onions, rhubarb).
- There is a need to develop programs that teach community members how to cook traditional food (e.g. at community kitchens) and new food species (e.g. salmon, halibut, clams).
- Community greenhouses could be constructed in order to provide opportunities to learn how to produce, use and preserve the harvests.
- Community Action Research Team (CART) could be implemented in each community.
- Schools should recognize traditional camp and hunting skills as part of the school curriculum and develop a way to obtain credits for these activities.
- Efforts should be made to improve the learning and use of traditional language in ISR communities.
- The responsibility of parents in teaching their kids should be recognized.
- Success stories of families living healthy lifestyles could be reported in Tusaayaksat, the quarterly IRC printed magazine.
- Hands-on traditional ways of teaching and learning should be developed and promoted in order to provide people with experiential learning opportunities.
- There is a need to teach community members how to access funding sources in order to give them the possibilities to get resources to develop local initiatives.
- Evaluations of community programs could be developed as a way to highlight their successes.
- Youth fora could be developed to develop leadership.
- Infrastructure for community centres could be set up.
- School breakfast and lunch programs could be expanded with traditional foods.

#### Governance and policy

The participants viewed governance and policy issues as central in the process of empowerment. The following programs were identified to improve governance at all levels:

- Pilot projects could be developed to provide grants or loans to buy hunting, trapping and fishing equipment.
- Pilot projects could be developed to support the use and maintenance of technology and equipment.
- Funding administration and reporting requirements should be adapted to accommodate the needs of the communities; funding programs and opportunities should be more flexible, multi-year, on-going, carry over.
- Local airlines could be requested to provide in-kind funding or seats to fly community members and youth between communities for health capacity building activities.
- Lobbying of all levels of leadership should be done to increase awareness about Inuit health, well-being, and food security issues.
- Changes in regional and local housing policy should be made to support housing and food security.
- Intercommunity trade could be promoted.
- Strategies and policies to attract and keep health professionals should be developed.
- Community-specific policy for timing of work and schooling should be developed to allow both adults and youth to go on the land during hunting season.
- Food security issues need to be considered in the development of wildlife management policies.

#### Research

Participants agreed that more research is needed in order to address the needs of ISR communities as well as the gaps in knowledge. The following research guidelines were suggested:

- Research topics and projects should be generated by communities and include local co-investigators, for example, CART.
- Research with most recent data should be continued in order to identify linkages between disease and food intake and lifestyle choices.
- Community-based monitoring should be implemented to enhance infrastructure.
- Research should investigate elements of success and non-success among previous health promotion initiatives in order to identify the factors that determine local factors of success.
- Research should investigate issues on the economics associated with traditional food substitution.
- Research should investigate the relationship between food availability, nutritional needs and harvest numbers in each community in order to ensure food security.
- The available research data should be used to support action and policy at the local and regional levels.

#### Housing

Housing was described as a major factor influencing food security, and various activities were identified to improve housing conditions and to promote food security:

- Organizations (national and international) that have programs promoting the building of affordable housing and the promotion of homeownership could become involved in the circumpolar region (e.g. Habitat for Humanity).
- Housing programs that have housing ownership as an endpoint should be developed, and housing policies should reflect the local needs of the communities.
- Long-term care facilities for elders should be improved to allow them to stay in the communities.
- Housing should be made more affordable and available.
- Housing conditions should be improved (e.g. mould issues).
- The testing of permafrost in communities should be implemented in order to determine how community infrastructure could be impacted by climate change.
- It should be recognized that households with multiple families face significant challenges regarding food security.

## Discussion and recommendations

Results from the 2007–2008 IHS have demonstrated that food insecurity and economic hardship, including food insecurity, nutrition transition and inadequate housing, represent fundamental public health challenges, which need to be addressed (3). In addition, the CCA Expert Panel on northern food security recently concluded that further research is required to reveal the health implications associated with the nutrition transition (2). During this workshop in Inuvik, lifestyle, chronic diseases, food insecurity and housing issues were central in the discussions concerning food security, health and well-being. These many issues cannot be dissociated from the ecological, social, political and economic changes that are currently occurring in the Arctic. In order to address these issues effectively, the empowerment of Inuit communities in the ISR was identified as a key to promote health and well-being and environmental sustainability.

A key theme of the workshop discussions focused on the issues of harvest support and traditional food sharing. Presently, all around the circumpolar area, Indigenous peoples rely on traditional foods for their subsistence (14). Traditional foods are of critical importance for the health and well-being of the Inuit, as they constitute important sources of energy, protein and nutrients, and they are central to the Inuit culture and identity (15, 16). Harvest support and traditional food sharing initiatives in the ISR could thus contribute to promote access to traditional food, thus promoting health and well-being of its people.

The discussions of the workshop also demonstrated that the participation of communities in the design of education and health promotion programs is a condition to their empowerment. Most issues raised during these discussions were about ensuring food security, with a particular focus on traditional foods. It has been shown that the determinants of food insecurity can operate at different spatial and temporal scales: food affordability and budgeting, food knowledge and preferences, food quality and availability, environmental stress, declining hunting activity and the cost of harvesting (17). Therefore, education and promotion programs should address these determinants, with active participation from Inuit communities.

Governance and policy were also an important issue raised by the participants of the workshop. Governance has been defined as the way governments and other social organizations interact, relate to citizens and take decisions in an increasingly complex world (18). Participants mentioned that regional and local policies should be developed to address food security, housing, retention of professionals in communities and funding administration. A recent scoping study showed that health promotion programs were successful only when they were initiated, designed and carried out under Aboriginal authority and leadership (19). In addition, the CCA panel of experts on northern food security suggested that there is a need to further understand the interconnected relationships among local, regional, and national levels of governance that support and facilitate action on climate change, food security and health (2).

Although housing was not initially addressed in the food safety and food security results presented by the researchers, participants from Inuit communities and organizations identified this issue as a major concern. Indeed, participants claimed that housing costs for rent or mortgage constituted a major factor hindering food security. In addition, participants mentioned that secure, adequate, safe housing was needed to store and prepare healthy foods.

The CCA report also stressed the importance of more local ownership in research processes and suggested that local research capacities need to be given more attention (2). Community participation is necessary to understand health and well-being, as Aboriginal peoples have “the deep knowledge, skills, experience, and understanding of local and broader community history, contemporary strengths, aspirations, ideas, experiences, resources and needs that form the bedrock upon which all effective health promotion is based” (19). Participants emphasized the fact that research results had to be translated into concrete actions to improve health and well-being.

The on-going development of local research capacities increases communities’ knowledge and understanding of their issues, and also contributes to engage them in finding local solutions to issues they may know intimately (2). It is recommended that future research and intervention projects be based on the issues discussed during this workshop. Health and food security-related projects and activities should use the vision, goals and actions identified in this workshop as the foundation for future work. Participants agreed to use the summary of the discussions as a reference document for future research and intervention projects, and to maintain the interdisciplinary and multi-sectorial approach in the development of the research and intervention projects. Community involvement is a key element in developing future research in the ISR.

## References

[1] Bjerregaard P, Young TK, Dewailly E, Ebbesson SO (2004). Indigenous health in the Arctic: an overview of the circumpolar Inuit population. Scand J Publ Health.

[2] CCA (2014). Aboriginal Food Security in Northern Canada: an Assessment of the State of Knowledge.

[3] Egeland GM (2011). Inuit Health Survey speaks to need to address inadequate housing, food insecurity and nutrition transition. Int J Circumpolar Health.

[4] Saudny H, Leggee D, Egeland G (2012). Design and methods of the Aduld Inuit Health Survey 2007–2008. Int J Circumpolar Health.

[5] GNWT Inuvialuit Comprehensive Land Claim Agreement 2008 [cited 2014 Jun 6].

[6] Statistics Canada Population and dwelling counts, for Canada, provinces and territories, and census subdivisions (municipalities), 2011 and 2006 censuses (table). Population and Dwelling Count Highlight Tables.

[7] Egeland GM, Johnson-Down L, Cao ZR, Sheikh N, Weiler H (2011). Food insecurity and nutrition transition combine to affect nutrient intakes in Canadian arctic communities. J Nutr.

[8] Rosol R, Huet C, Wood M, Lennie C, Osborne G, Egeland GM (2011). Prevalence of affirmative responses to questions of food insecurity: International Polar Year Inuit Health Survey, 2007–2008. Int J Circumpolar Health.

[9] Huet C, Rosol R, Egeland GM (2012). The prevalence of food insecurity is high and the diet quality poor in Inuit communities. J Nutr.

[10] Health Canada (2012). Household food insecurity in Canada in 2007–2008: key statistics and graphics.

[11] Galloway T, Blackett H, Chatwood S, Jeppesen C, Kandola K, Linton J (2012). Obesity studies in the circumpolar Inuit: a scoping review. Int J Circumpolar Health.

[12] Donaldson SG, Van Oostdam J, Tikhonov C, Feeley M, Armstrong B, Ayotte P (2010). Environmental contaminants and human health in the Canadian Arctic. Sci Total Environ.

[13] O'Neil JD, Elias B, Yassi A (1997). Poisoned food: cultural resistance to the contaminants discourse in Nunavik. Arctic Anthropol.

[14] Hansen JC (1998). The human health programme under AMAP. AMAP Human Health Group. Arctic monitoring and assessment program. Int J Circumpolar Health.

[15] Van Oostdam J, Donaldson SG, Feeley M, Arnold D, Ayotte P, Bondy G (2005). Human health implications of environmental contaminants in Arctic Canada: a review. Sci Total Environ.

[16] Nancarrow TL, Chan HM (2010). Observations of environmental changes and potential dietary impacts in two communities in Nunavut, Canada. Rural Rem Health.

[17] Ford JD, Beaumier M (2011). Feeding the family during times of stress: experience and determinants of food insecurity in an Inuit community. Geogr J.

[18] Plumptre T, Graham J (1999). Governance and good governance: international and aboriginal perspectives.

[19] Wise M, Angus S, Harris E, Parker S (2012).

